# Honey as Source of Nitrogen Compounds: Aromatic Amino Acids, Free Nucleosides and Their Derivatives

**DOI:** 10.3390/molecules25040847

**Published:** 2020-02-14

**Authors:** Piotr M. Kuś

**Affiliations:** Department of Pharmacognosy and Herbal Medicines, Faculty of Pharmacy, Wroclaw Medical University, ul. Borowska 211a, 50-556 Wrocław, Poland; kus.piotrek@gmail.com or piotr.kus@umed.wroc.pl

**Keywords:** essential and non-essential nutrients, nucleosides, honey composition, uridine, neuropharmacological activities

## Abstract

The content of selected major nitrogen compounds including nucleosides and their derivatives was evaluated in 75 samples of seven varieties of honey (heather, buckwheat, black locust, goldenrod, canola, fir, linden) by targeted ultra-high performance liquid chromatography-diode array detector - high-resolution quadrupole time-of-flight mass spectrometry (UHPLC-DAD-QqTOF-MS) and determined by UHPLC-DAD. The honey samples contained nucleosides, nucleobases and their derivatives (adenine: 8.9 to 18.4 mg/kg, xanthine: 1.2 to 3.3 mg/kg, uridine: 17.5 to 51.2 mg/kg, guanosine: 2.0 to 4.1 mg/kg; mean amounts), aromatic amino acids (tyrosine: 7.8 to 263.9 mg/kg, phenylalanine: 9.5 to 64.1 mg/kg; mean amounts). The amounts of compounds significantly differed between some honey types. For example, canola honey contained a much lower amount of uridine (17.5 ± 3.9 mg/kg) than black locust where it was most abundant (51.2 ± 7.8 mg/kg). The presence of free nucleosides and nucleobases in different honey varieties is reported first time and supports previous findings on medicinal activities of honey reported in the literature as well as traditional therapy and may contribute for their explanation. This applies, e.g., to the topical application of honey in herpes infections, as well as its beneficial activity on cognitive functions as nootropic and neuroprotective, in neuralgia and is also important for the understanding of nutritional values of honey.

## 1. Introduction

Nutrient and medicinal properties of honey have been appreciated since prehistoric times and the knowledge about its values is currently deepened and rediscovered. Honey is not only a source of simple carbohydrates, but also microelements, vitamins, antioxidants, prebiotics, and probiotic bacteria [1,2,3]. Recently, Begum et al. reported the presence of essential elements like K, Ca, Mn, and Fe in honey along with *Gluconobacter oxydans* that was found to possess probiotic properties with siderophorogenic potential [2]. Other research demonstrated that buckwheat honey increases antioxidant potential of serum, thus possibly could exert antioxidant-related health benefits [4]. Honey is also a good source of carbohydrates supporting during physical exercise and characterized by low glycemic index [3]. One of the specific groups of compounds in honey are those containing nitrogen, which is an essential component of basic compounds in plants such as nucleic acids, proteins, coenzymes, hormones, some vitamins, and chlorophylls. In honey, nitrogen compounds constitute a versatile group present as a minor compounds. The amino acids are found in all honey types in various proportions, depending on their floral source. The differences in their levels were useful to determine the botanical origin in combination with chemometrics [5]. Another, common groups of nitrogen compounds found in honey were water-soluble vitamins such as riboflavin, niacin, nicotinic acid, pantothenic acid or folic acid [6]. Some of the honey varieties contain relevant amounts of different classes of nitrogen compounds that may be useful as a specific or non-specific markers of botanical origin, for example purine alkaloids in *Coffea* spp. honey [7] or high amounts of kynurenic acid in *Castanea sativa* Mill. honey [8]. The latter was found to possess anti-scarring activity as well as downregulating IL-17/1L-23 axis *in vitro*, and this could reduce inflammation in autoimmune diseases such as psoriasis or alopecia [9,10]. Oelschlaegel et al. beside kynurenic acid, reported the presence of its metabolite 4-hydroxyquinoline in cornflower (*Centaurea cyanus* L.) honey [11]. Recently, we reported the presence of 5-*epi*-lithospermoside, a noncyanogenic cyanoglucoside as a marker of *Phacelia tanacetifolia* Benth. honey as well as the occurrence of nucleoside uridine and nucleobase—adenine as well as other purine—xanthine [12]. Nucleobases that are part of nucleosides and further—nucleotides, play fundamental role in construction of DNA and RNA. Those compounds may also have importance for nutritional values of honey, especially uridine that was found to play relevant role, e.g., in cognitive functions [13] as well as guanosine, that exhibits neurotrophic and neuroprotective effects interacting with glutamatergic and adenosinergic systems and calcium-activated potassium channels [14]. Not much is known on occurrence and content of nucleosides in different honey varieties. Therefore, the scope of the current study was targeted ultra-high performance liquid chromatography-diode array detector-high-resolution quadrupole time-of-flight mass spectrometry (UHPLC-DAD-QqTOF-MS) and UHPLC-DAD analyses of the most common types of Polish honeys to evaluate the content of selected nitrogen compounds, including nucleobases and nucleosides.

## 2. Results and Discussion

**Figure 1:**
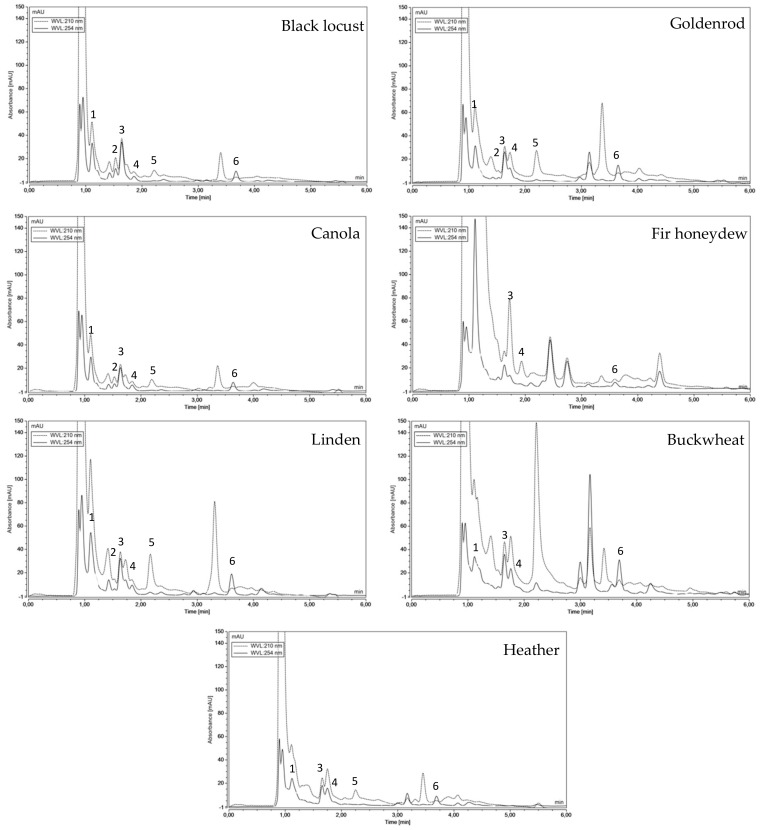
Representative UHPLC chromatographic profiles of different Polish unifloral honeys at λ = 210 nm and λ = 254 nm.

Chemical profiles of 75 samples of seven unifloral honey varieties were investigated by targeted UHPLC-DAD and UHPLC-DAD-QqTOF-MS analyses. Six major nitrogen compounds (Adenine Xanthine, Uridine, Tyrosine, Phenylalanine, Guanosine) were identified (Table 1) and quantified (Table 2). The comparison of chromatographic profiles obtained for honey varieties is presented on Figure 1 and demonstrates visible differences. All the mentioned compounds were identified by retention time, UV-Vis spectra and comparison with commercially available standard compounds as well as HRMS. For uridine, beside the main pseudomolecular ion 245.0764 [M + H]^+^, also 113.0346 [M − 132 + H]^+^, corresponding to uracil was present. The 132 amu loss corresponds to an unmodified ribose from the ribonucleoside, thus confirming nitrogen-carbon glyosidic bond. Similarly, guanosine was accompanied by 152.0570 [M − 132 + H]^+^ fragment, corresponding to a pseudomolecular ion of guanine.

The mean amount of adenine in different honey varieties ranged from about 8.9 to 18.4 mg/kg and was highest in linden (*Tilia* spp.) and lowest in canola (*Brassica napus* L.) honey. Xanthine and guanosine levels were quite low, and ranged from 1.2 in goldenrod (*Solidago* spp.) to 3.3 mg/kg in black locust and 2.0 to 4.1 mg/kg, respectively. Xanthine was not detected by UHPLC-DAD in heather [*Calluna vulgaris* (L.) Hull], buckwheat (*Fagopyrum esculentum* Moench) and fir (*Abies alba* Mill.) honeys. Guanosine in buckwheat and fir honey as well as adenine in the latter honey were not detected as well, due to the presence of overlapping peaks. Mean uridine content ranged from about 17.5 to 51.2 mg/kg and was lowest in canola and highest in black locust (*Robinia pseudoacacia* L.) honeys. The mean content of aromatic amino acids ranged from 7.8 to 263.9 and from 9.5 to 64.1 mg/kg for tyrosine and phenylalanine, respectively. The lowest amounts of tyrosine and phenylalanine were found in canola honey and the highest in buckwheat honey. Previously analyzed *P. tanacetifolia* honey contained higher amounts of adenine, with mean value 18.5 mg/kg almost equal to that found in linden honey. It contained also relatively high amount of uridine (42.8 mg/kg) similar to that found in buckwheat honey but it was also much richer in xanthine (mean value 10.5 mg/kg) than any other investigated variety [1].

The content of quantified compounds significantly differed between some of the varieties, suggesting strong link to the botanical origin. This indicates that some varieties such as black locust or buckwheat honeys may be more valuable as sources of nutrients.

**Figure 2:**
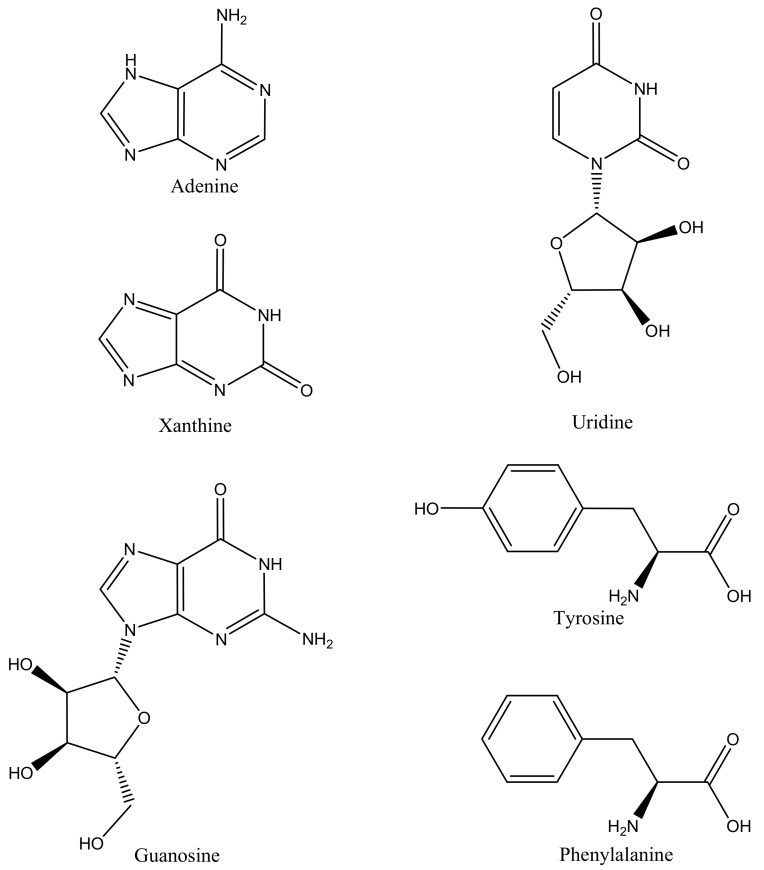
Structures of the nitrogen compounds determined in different honey varieties.

Adenine is a nucleobase (Figure 2) building nucleotides of the nucleic acids, as well as adenosine triphosphate (ATP), adenosine monophosphate (AMP), deoxy AMP, cyclic AMP, nicotinamide adenine dinucleotide (NAD) playing important roles in metabolism and signaling. Its derivatives possess also significant antiviral and cytostatic activity [2]. Xanthine commonly found in some plants is an adenosine degradation intermediate formed by oxidation of hypoxanthine [3]. Uridine and guanosine are RNA-specific nucleosides consisting of uracil (pyrimidine base) or guanine (purine base) and ribofuranose bound by β-N_1_ or β-N_9_ glycosidic bond, respectively. Uridine was found to act as nootropic, improving memory and learning ability as well as positively affecting mood. Dietary uridine was found to enhance the improvement in learning and memory of docosahexaenoic acid (DHA) fed gerbils [4] and enhanced synapse formation [5]. In combination with choline it improved cognitive deficits in rats [6]. Uridine, together with other key nutrients—omega-3 fatty acid DHA, and choline—accelerated the formation of a synaptic membrane and affected numbers of synapses formed initiated by neuronal activity [7]. Uridine decreased also depressive symptoms in patients with bipolar disorder and was patented as a treatment in doses starting from 1 g/day (Kondo et al., 2011; Renshaw, 2010). More recently, uridine at doses between 10 mg and 15 g per day was patented as prophylaxis and therapy of some tumors [8]. Interestingly, in double-blind, randomized, comparative, controlled trial, uridine in form of uridine triphosphate trisodium (1.5 mg, corresponding to 0.76 mg of uridine) in combination with cytidine monophosphate disodium (2.5 mg) and vitamin B_12_ (1 mg) administered 3 times a day showed superior improvement in pain reduction efficacy than vitamin B_12_ monotherapy of compressive neuralgia [9]. The amount of uridine corresponds to that present in 15–25 g of most honeys, thus moderate honey consumption (that contain free uridine) may be beneficial, also in prevention of this condition.

Guanosine is an extracellular signaling molecule exerting anti-inflammatory and antioxidative effects in several *in vivo* and *in vitro* injury models, modulates inflammation, downregulating of NFκB-mediated signaling [10]. It exhibits neurotrophic and neuroprotective effects interacting with glutamatergic, adenosinergic systems and calcium-activated potassium channels [11,12]. Guanosine provides also antidepressant-like effects [13] and attenuates behavioral deficits after traumatic brain injury by modulation of adenosinergic receptors [14]. Considering this, honey consumption may contribute to dietary prevention of different diseases. It may also partially explain and support previous reports on nootropic, memory-enhancing effects as well as neuropharmacological activities, such as antinociceptive, antidepressant but also neuroprotective potential of honey [15]. Guanosine is also known for antiviral activity against HSV-1, EC_50_ determined in infected Vero cells was 0.03 µM [16] and its analogues are widely used in treatment in herpesvirus infections [17]. Knowledge on concentration of guanosine in honey (approximately 5–10 µM), together with known wound-healing properties of honey [18], could support rationale of traditional treatment method of labial herpes by topical application of honey. Such beneficial activity was also confirmed in a small, non-blinded, cross-over study [19].

In general, the available literature data on content of phenylalanine and tyrosine, that are considered respectively essential and conditionally essential amino acids, in varietal honey is not very consistent [20,21,22,23] The mean contents of phenylalanine in Serbian black locust (acacia) honey and linden honey (8.5 and 9.2 mg/kg, respectively) as well as Polish black locust and heather honeys (6.4 and 16.0 mg/kg, respectively) were very similar to those found in current study. The values reported for canola (rapeseed), buckwheat and goldenrod honeys were much different (80.8, 10.4, 13.7 mg/kg, respectively). In case of tyrosine content, the results were similar to those reported for Serbian canola honey (8.9 mg/kg), but not to other data on Serbian or Polish varietal honeys [21,22]. Shen et al. found in buckwheat honey also a much higher amount of tyrosine than in other varieties which is consistent with our findings [23]. In fact, this compound was even proposed as marker of buckwheat honey [24]. From a nutritional point of view, the content of aromatic amino acids in honey is low.

**Figure 3:**
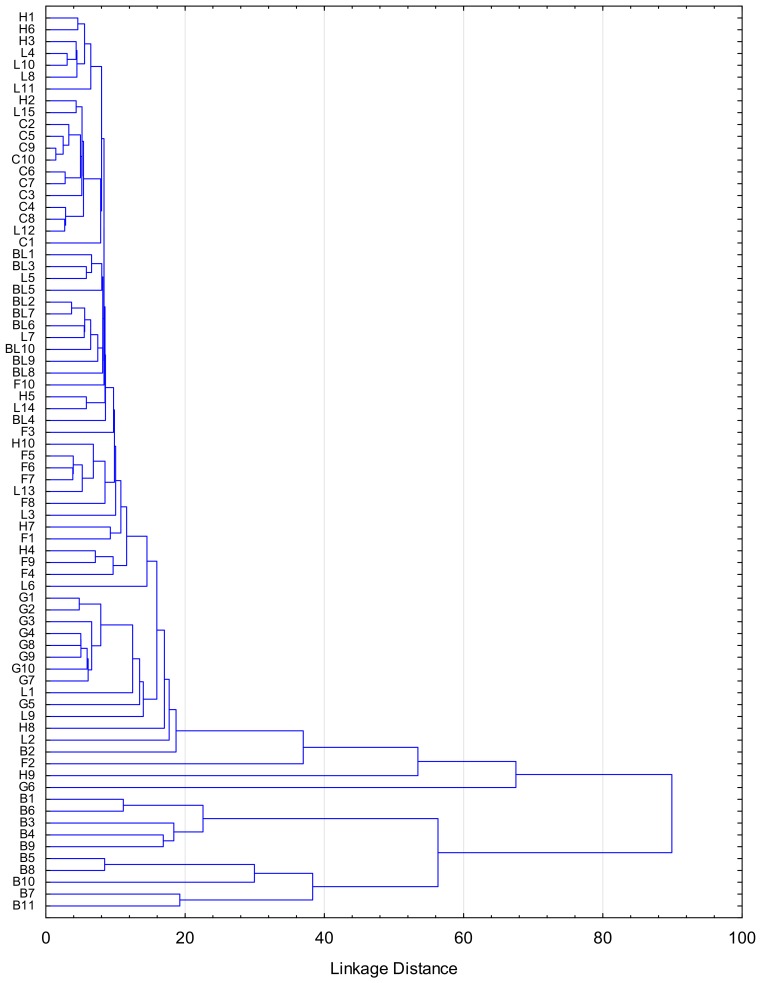
Dendrogram of different Polish unifloral honeys according to cluster analysis of similarity on the basis of the major compounds content (uridine, tyrosine, phenylalanine). H1-10—heather; B1-10—buckwheat; BL1-10—black locust; G1-10—goldenrod; C1-10—canola; F1-10—fir; L1-15—linden.

Some correlations between amounts of different components were found. The amount of adenine and guanosine was significantly, positively correlated within all dataset (R^2^ = 0.4818, *p* < 0.05). Amounts of tyrosine and phenylalanine were significantly correlated (*p* < 0.05) within the same some honey types (buckwheat, R^2^ = 0.7223; black locust, R^2^ = 0.6327; fir, R^2^ = 0.4607; linden, R^2^ = 0.5396; goldenrod, R^2^ = 0.9374). The levels of uridine and adenine were significantly correlated in goldenrod, R^2^ = 0.7421 and canola, R^2^ = 0.4651. The correlations between complementary nucleosides adenine and uridine could be explained by originating from RNA. The nucleobases as well as nucleosides may originate from plant, bee saliva or honey microbiome [25]. The significant differences found between various honey varieties suggest that plant origin may have the most important impact on their content. This is partially visibly also on a dendrogram demonstrating natural clustering of the samples, based on the major compounds (Figure 3). The linkage distance within and between groups indicates tendency for natural clustering and separation from other groups. It is particularly clear for buckwheat, black locust, goldenrod and canola honeys. The cluster containing buckwheat honeys demonstrates the biggest differences from the other samples. The balance between salvage and degradation of nucleotides helps to optimize the plant energy economy while maintaining levels of key elements. This includes nucleotide degradation, that involves removal of the phosphates from nucleotides to form nucleosides as well as cleavage of the nucleobase from the sugar mediated by nucleosidase allowing the base to be recycled as monophosphate [26]. The reason why these compounds would be excreted together with nectar is unknown. However, it is known that the components of nectar often protect against pathogens and modulate the behavior of nectar feeders, thus maximizing benefits for the plant [27].

## 3. Materials and Methods

### 3.1. Honey Samples

Seventy five samples of unifloral honey samples—heather [*Calluna vulgaris* (L.) Hull]–10, buckwheat (*Fagopyrum esculentum* Moench)–10, black locust (acacia) (*Robinia pseudoacacia* L.)–10, goldenrod (*Solidago* spp.)–10, canola (rapeseed) (*Brassica napus* L.)–10, fir honeydew (*Abies alba* Mill.)–10, linden (lime-tree) (*Tilia* spp.)–15. Honey samples were obtained from professional beekeepers in different parts of Poland in 2016–2018. The samples were stored in closed glass jars in dark, at 4 °C. Melissopalynological analyses were done according the International Commission for Bee Botany [28].

### 3.2. Reagents

Acetonitrile (gradient and LC-MS grade), LC-MS grade water, formic acid and phosphoric acid of analytical grade (Fluka™) were from Honeywell (Seelze, Germany), while adenine, guanosine and uridine standards were from Sigma-Aldrich (Steinheim, Germany). Tyrosine and phenylalanine were from Merck (Darmstadt, Germany) and xanthine from Reanal (Budapest, Hungary). Ultrapure H_2_O (<0.06 μS/cm) was obtained from Hydrolab HLP20UV (Hydrolab, Straszyn, Poland) device.

### 3.3. UHPLC-DAD and UHPLC-QqTOF-MS Analysis

The analyses were performed similarly as described previously [1]. In short, UHPLC-DAD Thermo Scientific™-Dionex™ system UltiMate™ 3000 fitted with a pump module, autosampler module, column thermostate and a diode array detector (Thermo Scientific™ Dionex™, Sunnyvale, CA, USA) was used. The system was set to record 3D data as well as set at 210 and 254 nm. The chromatographic separation was obtained on Phenomenex Kinetex^®^ EVO C18 110 Å column (150 mm × 2.10 mm, 2.6 µm, Phenomenex, Torrence, CA, USA) at 35 °C. The gradient was constructed using 0.2 M phosphoric acid in water (solvent A) and acetonitrile (solvent B) as a mobile phase at 0.4 mL/min. The gradient started with 100% of solvent A, decreased to 95% in 4 min, to 5% within 0.5 min, and remained at this concentration for 3 min. The system was washed and stabilized, before each injection (5 µL). The results were elaborated with a Chromeleon v7.2 SR5 software (Thermo Scientific™ Dionex™, Sunnyvale, CA, USA, 2017). Standard solutions were dissolved in methanol or water (xanthine), and diluted in ultrapure water and the calibrations curve concentrations ranged from approximately 0.2 to 30 mg/L (correlation values 0.9999–1.0000). Before analysis, the honey samples were dissolved in ultrapure water (1:5, *w*/*v*) and filtered through H-PTFE membrane (0.2 µm, Ø 25 mm, Macherey Nagel™, Düren, Germany). The limits of detection (LOD) and quantification (LOQ) were determined according the International Conference on Harmonization of Technical Requirements for Registration of Pharmaceuticals for Human Use (ICH) guidance note [29]. LOD and LOQ were calculated as following: LOD = 3.3 σ/S and LOQ = 10 σ/S, where σ is the standard deviation of blank and S is the slope of proper calibration plot.

UHPLC-QqTOF-MS analyses were performed in similar setting and conditions, except of solvent A, which was 0.1% formic acid in water. ESI-HRMS analysis was performed with Compact™ QqTOF mass spectrometer (Bruker Daltonic, Bremen, Germany). The following settings were applied as previously [1]: positive mode, the ion source temperature 100 °C, nebulizer gas pressure at 2.0 bar, dry gas flow 8.0 L/min and temperature 210 °C. The capillary voltage was set at 4.50 kV. The collision energy was set on 8.0 eV and for MS/MS: 35 eV. Sodium formate clusters at concentration 10 mM were used for internal calibration.

### 3.4. Statistical Analysis

Statistical analyses were performed using the Statistica 64 v13.1 software (StatSoft Inc., Tulsa, OK, USA) and R for Windows, version 3.6.2 (R-Cran project, http://cran.r-project.org/). The results were expressed as the mean ± SD and analyzed by ANOVA followed by the Tukey test. The Pearson’s product-moment correlation was used to assess relationships between the parameters and their significance was assessed in two-tailed test at *p* < 0.05. The variations of parameters among different honey samples were evaluated by cluster analysis (hierarchical-tree clustering). A dendrogram was used as an output of hierarchical clustering to show the hierarchical relationship between different samples and to find the best way to allocate them to clusters.

## 4. Conclusions

The targeted analysis revealed presence of adenine, xanthine, tyrosine, phenylalanine, and guanosine that varied between different honey types. The occurrence of nucleosides and nucleobases was found not to be specific only for a particular honey type. However, in some cases it significantly varied, indicating those more and less valuable as sources of nutrients. It may be useful for distinguishing or quality evaluation of some honey types. Honey may be considered as moderate dietary source of free uridine and black locust honey contains significantly higher amount of uridine (except buckwheat honey) than most of the investigated varieties. On the other hand, mean levels of adenine and guanosine were significantly more abundant in linden honey than in the most other varieties. The presence of nucleosides and nucleobases may partially explain the beneficial activities, properties reported in scientific literature and traditionally attributed to honey. This includes its topical application in herpes infections as well as its beneficial neurological activities: antinociceptive, nootropic, and neuroprotective among others.

## Figures and Tables

Column: Phenomenex Kinetex EVO C18 110 Å column (150 mm × 2.10 mm, 2.6 μm) eluted with mixtures of 0.2 mol/L phosphoric acid (solvent A) and acetonitrile (solvent B) at a flow rate of 0.4 mL/min. 1—adenine; 2—xanthine; 3—uridine; 4—tyrosine; 5—guanosine; 6—phenylalanine.

**Table 1:** Nitrogen compounds identified in the investigated honey samples.

	Compound	t_R_ (min)	UV_max_ (nm)	[M + H]^+^	Formula	Error (ppm)
1	Adenine	1.17	263	136.0621	C_5_H_5_N_5_	1.62
2	Uridine	1.72	262	245.0764	C_9_H_12_N_2_O_6_	3.93
3	Xanthine	1.78	268	153.0405	C_5_H_4_N_4_O_2_	4.91
4	Tyrosine	2.21	224, 275	182.0812	C_9_H_11_NO_3_	2.75
5	Guanosine	3.10	254, 274	284.0989	C_10_H_13_N_5_O_5_	2.09
6	Phenylalanine	3.41	258	166.0864	C_9_H_11_NO_2_	2.41

**Table 2:** Amounts of the identified nitrogen compounds in varietal honey samples.

	Adenine (mg/kg)	Xanthine (mg/kg)	Uridine (mg/kg)	Tyrosine (mg/kg)	Guanosine (mg/kg)	Phenylalanine (mg/kg)
**Heather**	11.8^a^ ± 2.9	*nd*	30.7^bc^ ± 9.9	35.8^a^ ± 28.9	3.4^ab^ ± 1.1	17.1^abc^ ± 4.0
*(n = 10)*						
**Buckwheat**	11.6^a^ ± 4.4	*nd*	40.2^cd^ ± 13.6	263.9^b^ ± 91.6	nd*	34.6^c^ ± 17.1
*(n = 10)*						
**Black locust**	11.1^a^ ± 1.5	3.3^b^ ± 0.8	51.2^d^ ± 7.8	12.1^a^ ± 5.2	2.6^a^ ± 0.3	11.3^ab^ ± 5.5
*(n = 10)*						
**Goldenrod**	14.0^ab^ ± 2.7	1.2^a^ ± 0.6	24.6^ab^ ± 2.7	44.9^a^ ± 25.1	2.5^a^ ± 1.3	64.1^d^ ± 20.3
*(n = 10)*						
**Canola**	8.9^a^ ± 1.4	3.0^b^ ± 0.6	17.5^a^ ± 3.9	7.8^a^ ± 3.3	2.0^a^ ± 0.7	9.5^a^ ± 3.6
*(n = 10)*						
**Fir**	nd*	*nd*	32.5^bc^ ± 12.8	31.6^a^ ± 17.9	nd*	18.1^abc^ ± 13.3
*(n = 10)*						
**Linden**	18.4^b^ ± 6.5	1.7^a^ ± 0.9	28.6^ab^ ± 10.6	21.8^a^ ± 12.3	4.1^b^ ± 1.5	28.4^bc^ ± 20.6
*(n = 15)*						
LOD	0.1	0.2	0.3	0.4	0.1	0.3
LOQ	0.4	0.6	1.0	1.1	0.3	0.8

The amount of compounds is expressed in milligrams per kilogram of honey as average (AV) ± standard deviation (SD); *nd*—not detected; *—overlapping peak; the mean values with different letters within the same column differ significantly at *p* < 0.05; *n*—number of samples; LOD—limit of detection; LOQ—limit of quantification.
